# Acute Zika Virus Infection in an Endemic Area Shows Modest Proinflammatory Systemic Immunoactivation and Cytokine-Symptom Associations

**DOI:** 10.3389/fimmu.2018.00821

**Published:** 2018-05-03

**Authors:** Jéssica Barletto de Sousa Barros, Paulo Alex Neves da Silva, Rosemary de Carvalho Rocha Koga, Patrícia Gonzalez-Dias, José Rodrigues Carmo Filho, Patrícia Resende Alo Nagib, Verônica Coelho, Helder I. Nakaya, Simone Gonçalves Fonseca, Irmtraut Araci Hoffmann Pfrimer

**Affiliations:** ^1^Department of Master in Environmental Sciences and Health, School of Medical, Pharmaceutical and Biomedical Sciences, Pontifical Catholic University of Goiás, Goiânia, Brazil; ^2^Department of Microbiology, Immunology, Parasitology and Pathology, Institute of Tropical Pathology and Public Health, Federal University of Goiás, Goiânia, Brazil; ^3^Department of Pathophysiology and Toxicology, School of Pharmaceutical Sciences, University of São Paulo, São Paulo, Brazil; ^4^Laboratory of Immunology, Heart Institute (InCor), School of Medicine, University of São Paulo, São Paulo, Brazil; ^5^Institute for Investigation in Immunology – National Institute of Science and Technology – iii – INCT, São Paulo, Brazil

**Keywords:** acute Zika virus infection, cytokines, chemokines, symptoms, immune response, low viremia, immunoactivation

## Abstract

An early immune response to Zika virus (ZIKV) infection may determine its clinical manifestation and outcome, including neurological effects. However, low-grade and transient viremia limits the prompt diagnosis of acute ZIKV infection. We have investigated the plasma cytokine, chemokine, and growth factor profiles of 36 individuals from an endemic area displaying different symptoms such as exanthema, headache, myalgia, arthralgia, fever, hyperemia, swelling, itching, and nausea during early-phase infection. These profiles were then associated with symptoms, revealing important aspects of the immunopathophysiology of ZIKV infection. The levels of some cytokines/chemokines were significantly higher in acute ZIKV-infected individuals compared to healthy donors, including interferon (IFN) gamma-induced protein 10 (IP-10), regulated on activation, normal T cell expressed and secreted (RANTES), IFN-γ, interleukin (IL)-9, IL-7, IL-5, and IL-1ra, including some with predominantly immunoregulatory activity. Of note, we found that higher levels of IP-10 and IL-5 in ZIKV-infected individuals were strongly associated with exanthema and headache, respectively. Also, higher levels of IL-1ra were associated with subjects with arthralgia, whereas those with fever showed lower levels of granulocyte-colony stimulating factor (G-CSF). No correlation was observed between the number of symptoms and ZIKV viral load. Interestingly, only IP-10 showed significantly decreased levels in the recovery phase. In conclusion, our results indicate that acute ZIKV infection in a larger cohort resident to an endemic area displays a modest systemic immune activation profile, involving both proinflammatory and immunoregulatory cytokines and chemokines that could participate of virus control. In addition, we showed that differential cytokine/chemokine levels are related to specific clinical symptoms, suggesting their participation in underlying mechanisms.

## Introduction

Zika virus (ZIKV) infection is an emerging endemic disease, with recent outbreaks in the Americas (1). Approximately 80% of ZIKV infections are asymptomatic, while symptomatic cases present similar clinical features to dengue and chikungunya fevers, and are characterized mainly by exanthema, fever, headache, arthralgia, myalgia, and conjunctivitis (2–5). However, in the recent outbreaks, adult ZIKV infection was associated with Guillain–Barré syndrome (6), myelitis, meningoencephalitis (7, 8), and a fetal neurological syndrome involving microcephaly (9–11). ZIKV has been detected in fetal brain tissue, suggesting neurotropism (12), and in body fluids such as blood, urine, saliva, tears, and semen for >6 months, suggesting viral persistence and dissemination (13).

Due to the absence of symptoms or presence of mild ones, little is known about the early immune response to ZIKV infection in humans. *In vitro* studies have demonstrated that many different cell types, including glomerular cells (14), retinal endothelial cells (15, 16), brain microvascular endothelial cells (17), dendritic cells (18), and monocytes (19, 20) can be infected by ZIKV. Changes in pro- and anti-inflammatory cytokines have been reported in a small group of six individuals, who were infected with ZIKV while visiting endemic areas (21). Considering the small number of patients in that study, an evaluation of immune mediators in the acute phase in a larger cohort of patients residing in an endemic area may shed some light on the immunopathophysiology of ZIKV infection.

In this work, we investigated the levels of a broad array of cytokines, chemokines, and growth factors during acute ZIKV infection in an endemic area in Brazil. We examined the levels of 27 immune-active molecules with distinct functions, including pro- and anti-inflammatory cytokines with Th1, Th2, Th17, Th9, and Treg cell expression patterns, as well as chemokines, and growth factors involved in innate and adaptive immune responses. Only seven cytokines displayed higher levels in acutely infected patients compared to healthy individuals. We found significantly higher levels of IL-1ra in ZIKV-infected individuals with arthralgia and IL-5 in those with headache. Interestingly, higher levels of interferon (IFN) gamma-induced protein 10 (IP-10) were associated with exanthema, whereas lower levels of granulocyte-colony stimulating factor (G-CSF) were detected in subjects with fever, suggesting that cytokines can either induce or control the clinical symptoms of ZIKV infection.

## Materials and Methods

### Ethics Statement

Ethical approval for this study was obtained from the Institutional Review Board of Pontifical Catholic University of Goiás (CEP—Research Ethics Committee), under the protocol number 46073815.9.0000.00370. All study subjects signed a written informed consent form before the interview, and blood and urine collection was performed in accordance with the Declaration of Helsinki.

### Study Participants and Sample Collection

Our study cohort included 36 individuals aged 18–68 years (median 36 years), from Goiânia, Goiás, Brazil, who developed acute Zika-like symptoms during the Brazilian ZIKV outbreak in January–May 2016, and had a ZIKV diagnosis confirmed by real-time reverse transcription-polymerase chain reaction (RT-PCR). Eight of the 36 ZIKV-infected subjects (22.2%) were males and 28 (77.8%) were females (Table 1). Subjects were eligible to participate in this study if they were at least 18 years old. Subjects positive for dengue and chikungunya by RT-PCR were excluded. As controls, 28 healthy potential blood donors, aged 18–59 years (median 33 years) and displaying negative blood tests for several infectious diseases, were recruited from the Center of Serology and Immunohematology of Goiânia, Brazil. All subjects were invited to participate in the study after being provided an explanation of the research, and all signed a written informed consent form. ZIKV-infected subjects were interviewed in a private room and answered a written questionnaire regarding the day of symptom onset, types of symptoms, and demographic features. Blood collection from ZIKV-infected subjects was performed at enrollment, 1–15 days after symptom onset. Urine samples were also collected from the ZIKV-infected group. Six ZIKV-infected subjects underwent a second blood collection 2–3 weeks after the first sample to evaluate the recovery phase. Blood samples were collected into ethylenediaminetetraacetic acid-coated Vacutainer tubes (Becton & Dickinson, USA), and the plasma was separated and stored at −80°C until analysis. Specimens from indivi-duals with symptoms of ZIKV infection were handled using biosafety level 2 precautions, following all the safety criteria indicated by the precaution standards of the Healthcare Infection Control Practices Advisory Committee and described in the Centers for Disease Control and Prevention/National Institutes of Health publication Biosafety in Microbiological and Biomedical Laboratories.^1^

**Table 1:** Characteristics of Zika virus (ZIKV)-infected subjects.

Subject code	Sex^a^	Age (years)	Days (symptom onset to sampling)	qPCR ZIKV (C.T.)^b^
1	F	34	NA^c^	30.94
2	F	33	NA	32.49
3	M	36	NA	33.12
4	M	55	2	21.74
5	F	32	3	27.21
6	F	40	3	24.29
7	M	61	4	24.24
8	F	60	8	27.45
9	F	68	15	33.68
10	M	18	2	33.92
11	F	33	9	29.28
12	F	28	2	27.21
13	F	22	2	31.32
14	F	56	6	34.10
15	F	68	3	29.41
16	F	38	3	33.35
17	F	53	3	27.28
18	F	34	3	31.25
19	F	36	4	38.92
20	F	47	4	36.73
21	M	24	3	37.88
22	F	37	2	32.66
23	F	39	2	32.66
24	F	36	8	29.71
25	F	37	NA	29.20
26	M	18	6	32.05
27	F	34	2	33.19
28	F	25	6	27.42
29	F	42	4	31.77
30	F	30	2	31.06
31	F	23	8	33.16
32	M	56	4	31.77
33	F	36	2	35.85
34	F	49	4	35.82
50	F	28	4	35.33
51	M	18	5	38.79

*^a^Sex: M, male; F, female*.

*^b^CT, cycle threshold (<39)*.

*^c^NA, non-available*.

### ZIKV Real-Time RT-PCR

Viral RNA was obtained from plasma or urine using a QIAamp Viral RNA Mini Kit (Qiagen, Hilden, Germany), according to the manufacturer’s instructions. Real-time RT-PCR for ZIKV was performed using a kit (Bioclin^®^, Bio gene ZIKV PCR-K-203-6), with the following primers and probes: ZIKV-F, 5′-CCGCTGCCCAACACAAG-3′; ZIKV-R, 5′-CCACTAACGTTCTTTTGCAGACAT-3′; ZIKV-P, 5′-FAM-AGCCTACCTTGACAAGCAGTCAGACACTCAA-BHQ1-3′, developed according to Lanciotti et al. (22), with modifications. RT-PCR was performed following the manufacturer’s instructions. RT-PCR was also performed to detect the viruses causing chikungunya and dengue fevers.

### Cytokine, Chemokine, and Growth Factor Quantification

The quantitative detection of 27 cytokines [interferon (IFN)-γ, tumor necrosis factor (TNF)-α, IL-2, IL-4, IL-5, IL-6, IL-7, IL-9, IL-10, IL-12 (p70), IL-13, IL-15, IL-17, IL-1β, IL-1 receptor antagonist (IL-1ra), chemokines (MCP-1) (CCL2), MIP-1α (CCL3), MIP-1β (CCL4), RANTES (CCL5), IP-10 (CXCL10), IL-8 (CXCL8), eotaxin] and growth factors (basic FGF, G-CSF, GM-CSF, PGDF-BB, and VEGF), in the plasma of patients and healthy controls was performed using a commercial kit (Bio-Plex Pro Human Cytokine 27-Plex Immunoassay, BioRad) and a Luminex™ Instrumentation System (Bio-Plex Workstation from Bio-Rad Laboratories). The test was performed using 50 µL of plasma from all study subjects, according to the manufacturer’s instructions. Analyte concentrations were calculated using the software provided by the manufacturer (Bio-Plex Manager Software).

### Statistical Analysis

Differences in log2 cytokine levels between ZIKV-infected subjects and healthy controls were assessed using limma (23) with a significance level of *p* < 0.05 and a fold-change >2. Additionally, we used Mann–Whitney non-parametric analysis and rank product analysis (24) to compare the levels of plasma cytokines between ZIKV-infected subjects and healthy controls, and Chi-square tests to compare the detection frequency of each cytokine in ZIKV-infected subjects and healthy controls. The Spearman correlation test (*p* < 0.05 and *R* > 0.5) was used to identify associations between different cytokines/chemokines. To identify associations between cytokine levels and symptoms, we used Student’s *t*-test (*p*-value <0.05 and fold-change >2) to compare infected patients with and without specific symptoms. Random Forest analysis (25) was utilized to classify the importance of cytokines in predicting ZIKV infection and clinical symptoms (R package “randomForest”). The Wilcoxon matched-pair test was used to compare cytokine plasma levels in the same subjects in the acute and recovery phases (*p*-value < 0.05). We used GraphPad Prism 6.0 software (GraphPad Software, San Diego, CA, USA), R packages “heatmap2,” “corrplot,” and “ggplot” and Cytoscape^2^ to display the results.

## Results

### Acute ZIKV Infection Induces Changes in Only Seven Cytokines/Chemokines

**Figure 1:**
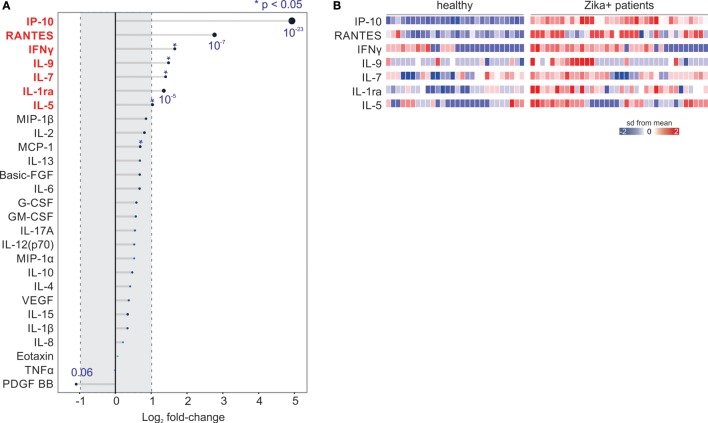
Cytokine/chemokine profiling in acute Zika virus (ZIKV) infection. **(A)** Differences in plasma cytokine/chemokine levels between acute ZIKV-infected and healthy subjects. Names in red represent cytokines with significantly higher levels upon infection (*p* < 0.05 and greater than twofold induction). The size of the circles is proportional to the significance. *p*-Values are shown in blue. **(B)** Individual levels of the cytokines upregulated in acute ZIKV infection. Each column represents a study subject. The levels of each cytokine/chemokine (rows) were *z*-normalized.

**Figure 2:**
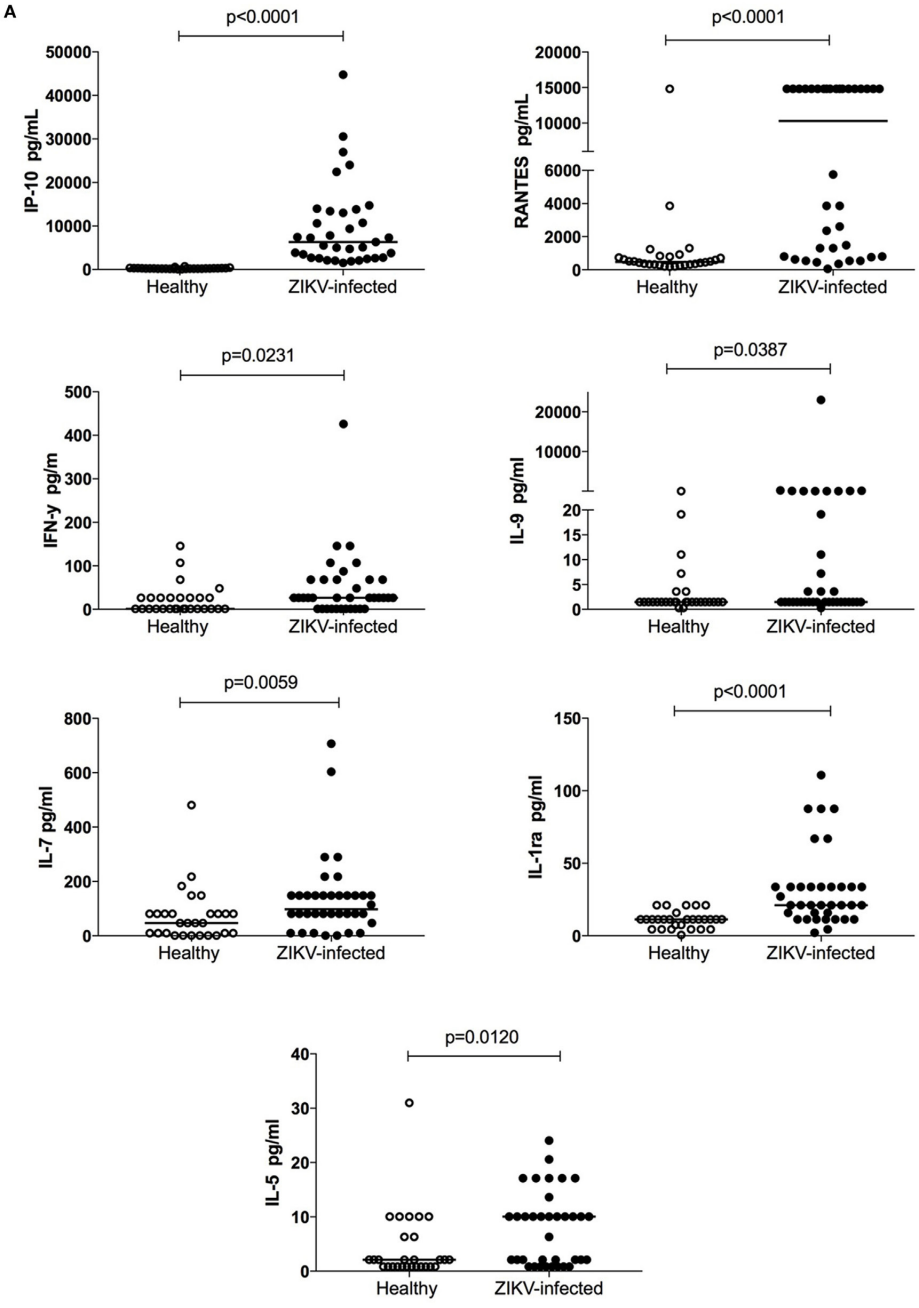

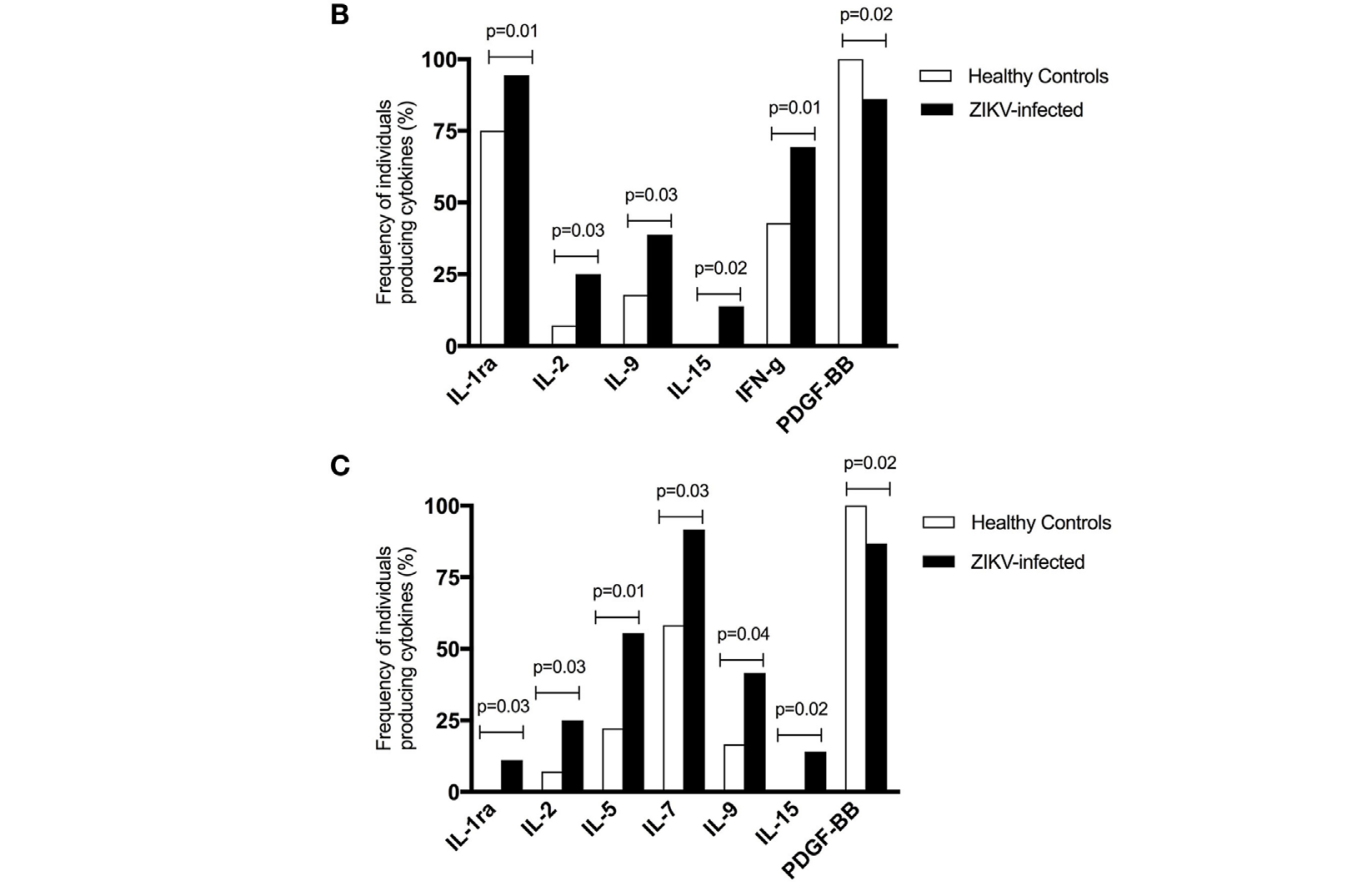
Plasma concentration and frequency of differentially expressed cytokines in acute Zika virus (ZIKV)-infected individuals compared to healthy controls. **(A)** Cytokines measured in the plasma of acute ZIKV-infected individuals and healthy controls and the concentrations of differentially expressed cytokines are shown. The dots on the graphs represent individuals. *p*-Values are indicated at the top of each graph. The non-parametric Mann–Whitney test was used and considered significant when *p* < 0.05. The cytokine frequencies of ZIKV-infected individuals and healthy controls showed significant differences considering the limit of detection of the test **(B)** and based on the standard curve range **(C)**. Chi-square tests were used **(B,C)** and *p* < 0.05 was considered significant.

To characterize early immune activation during acute ZIKV infection, we compared the plasma levels of cytokines, chemokines, and growth factors between 36 ZIKV-infected subjects and 28 healthy subjects. IP-10 (*p* < 0.0001) showed the highest levels in infected individuals compared to healthy subjects, followed by RANTES (*p* < 0.0001), IFN-γ (*p* = 0.02), IL-9 (*p* = 0.03), IL-7 (*p* = 0.005), IL-1ra (*p* < 0.0001), and IL-5 (*p* = 0.01; Figures 1A,B and 2A). We also tested the differences in cytokine plasma levels between infected patients and healthy subjects by rank product testing (24). Using this approach, the levels of three cytokines were higher in patients with infection: IP-10 (*p* < 0.001), RANTES (*p* < 0.001), and IFN-γ (*p* < 0.03). This confirmed the importance of these three cytokines in acute ZIKV infection.

Only five cytokines were more frequently detected in ZIKV-infected subjects compared to healthy controls, namely IL-1ra (*p* = 0.01), IL-2 (*p* = 0.03), IL-9 (*p* = 0.03), IL-15 (*p* = 0.02), and IFN-γ (*p* = 0.01). In contrast, PDGF-BB was more frequently detected in healthy subjects than in ZIKV-infected individuals (*p* = 0.02; Figure 2B). A similar pattern was observed when we calculated the frequencies of cytokine detection based on curve values, rather than on the detection limit of each cytokine. In this more stringent analysis, the frequency difference of IFN-γ was no longer significant, whereas IL-5 and IL-7 showed higher frequency of detection in acute ZIKV infection in relation to the normal physiological state (Figure 2C). We confirmed the Luminex assay with enzyme-linked immunosorbent assays for IFN-γ and IL-10 (Figure S1 in Supplementary Material). Finally, we found no correlations between ZIKV RNAemia levels and cytokine or chemokine plasma levels (data not shown).

### Cytokine and Chemokine Level Correlations in the Plasma of Acute ZIKV-Infected Subjects

**Figure 3:**
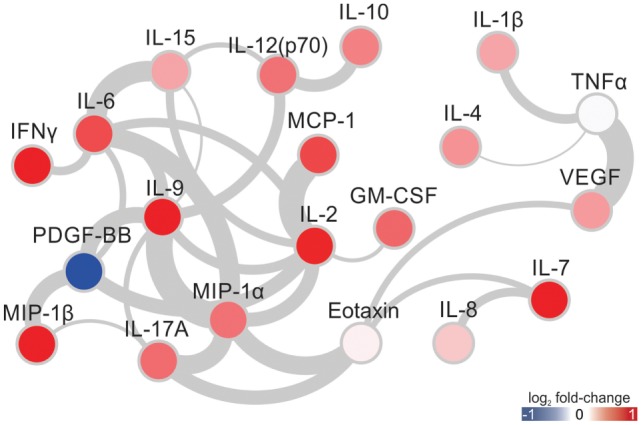
Cytokine/chemokine level correlations in Zika virus-infected subjects. Connections (edges) between two given cytokines represent direct significant correlations (Spearman *p*-value <0.05 and *R* > 0.5). The width of each edge is directly proportional to the *R* value. The node colors represent the fold-changes in cytokine/chemokine levels between infected and healthy subjects.

We next tested whether the plasma levels of individual cytokines in subjects with acute ZIKV-infection were related. We compared the levels of all cytokines with each other, and constructed a network representing these associations (Figure 3). Increased levels of IL-6 were associated with higher levels of IL-15, MIP-1α, IL-9, IL-2, IFN-γ, and PDGF-BB. IL-2 levels were correlated with the levels of MCP-1, PDGF-BB, MIP-1α, IL-9, and IL-15. MIP-1α strongly correlated with IL-9, followed by IL-17A and eotaxin. MIP-1β correlated with IL-17A. We also observed a direct correlation between the levels of MIP-1β and PDGF-BB, although the later has lower levels in infected individuals compared to healthy subjects. Moreover, TNF-α correlated with VEGF, IL-1β, and IL-4. Eotaxin levels were correlated with MIP-1α, IL-17A, VEGF, and IL-7. These associations show potential synergic effects of these cytokines, chemokines, and growth factors during acute ZIKV infection.

### Associations Between Cytokine/Chemokine Levels and Clinical Symptoms

**Figure 4:**
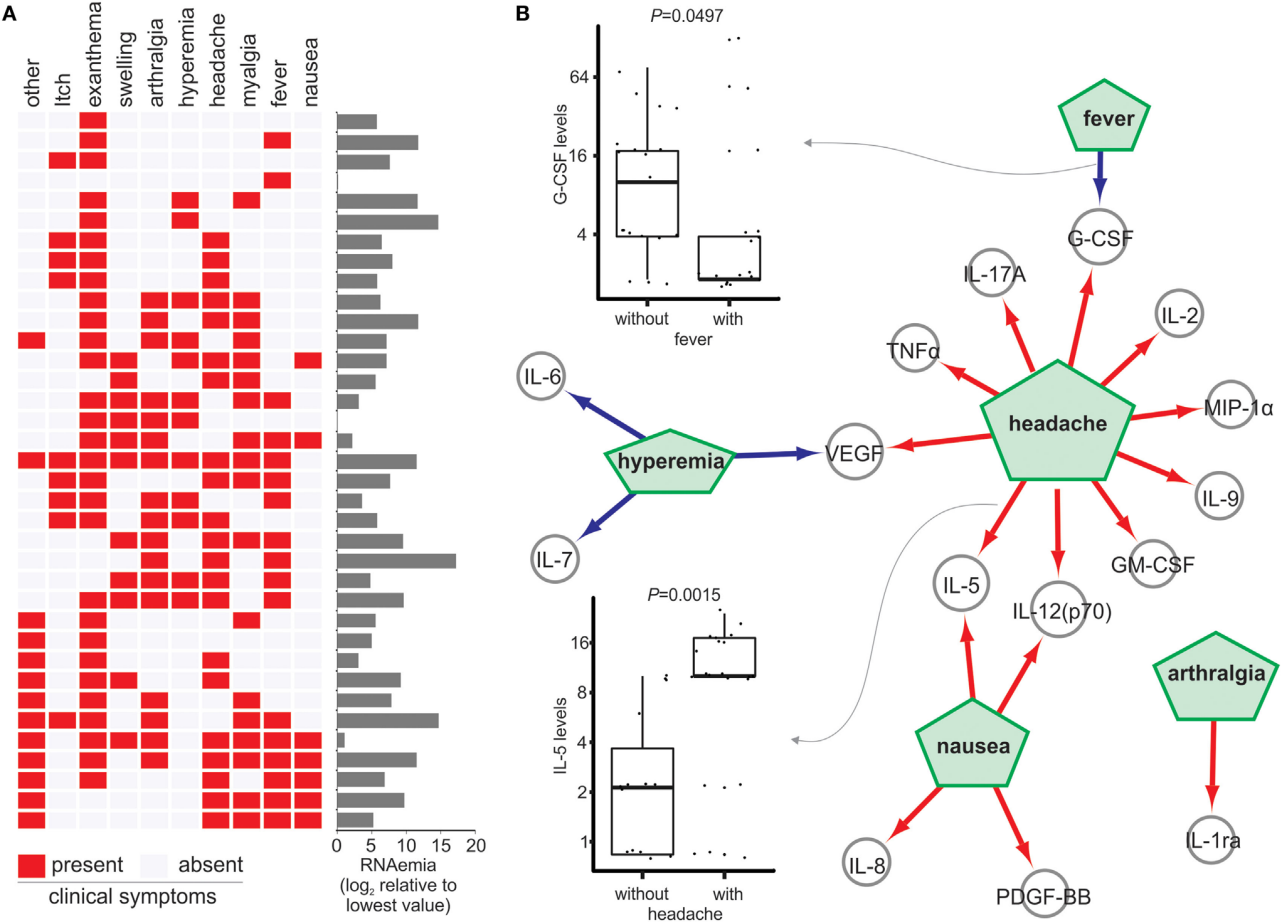
Cytokine and chemokine level changes associated with clinical symptoms. **(A)** Clinical symptoms in Zika virus (ZIKV)-infected subjects. The heat map represents the presence (red) or absence (gray) of symptoms (columns) in ZIKV-infected subjects (rows). RNAemia levels for each subject are shown in the bar graph. **(B)** Different cytokine levels were associated with different symptoms. The green pentagons represent clinical symptoms and the nodes represent the cytokines and the edges represent putative associations among the pentagons. Red arrows indicate that the level of a given cytokine is higher in ZIKV-infected subjects with the symptoms compared to subjects without them. Blue arrows indicate the cytokine level is lower in patients with the associated symptoms compared to those without them. The box plots show the levels of granulocyte-colony stimulating factor and IL-5 in subjects with fever and headache, respectively. Dots on the graphs represent individuals. *p*-Values are indicated at the top of each box plot.

The clinical symptoms of ZIKV-infected subjects during the acute phase are represented in a heat map in Figure 4A. The most frequent clinical symptoms observed were exanthema (80.5%), headache (58.3%), myalgia (50%), arthralgia (47.2%), fever (47.2%), hyperemia (33.3%), swelling (30.5%), itching (25%), and nausea (19.4%). All other symptoms were catagorized as “other” (36.1%). No correlation between the number of symptoms and ZIKV RNAemia was observed (Figure 4A).

Zika virus-infected subjects who reported headaches showed higher plasma levels of TNF-α, IL-17A, IL-2, IL-9, IL-12p70, MIP-1α, G-CSF, GM-CSF, and VEGF, and significantly higher levels of IL-5 (*p* = 0.0015; Figure 4B) compared to those without headache. Those with nausea showed higher plasma levels of IL-5, IL-12p70, IL-8, and PDGF-BB compared to those without nausea. Additionally, ZIKV-infected subjects with arthralgia showed higher levels of IL-1ra, whereas those with hyperemia showed lower plasma levels of IL-6, IL-7, and VEGF (Figure 4B). Finally, the plasma levels of G-CSF were significantly lower in ZIKV-infected subjects with fever than in those without fever (*p* = 0.0497; Figure 4B).

**Figure 5:**
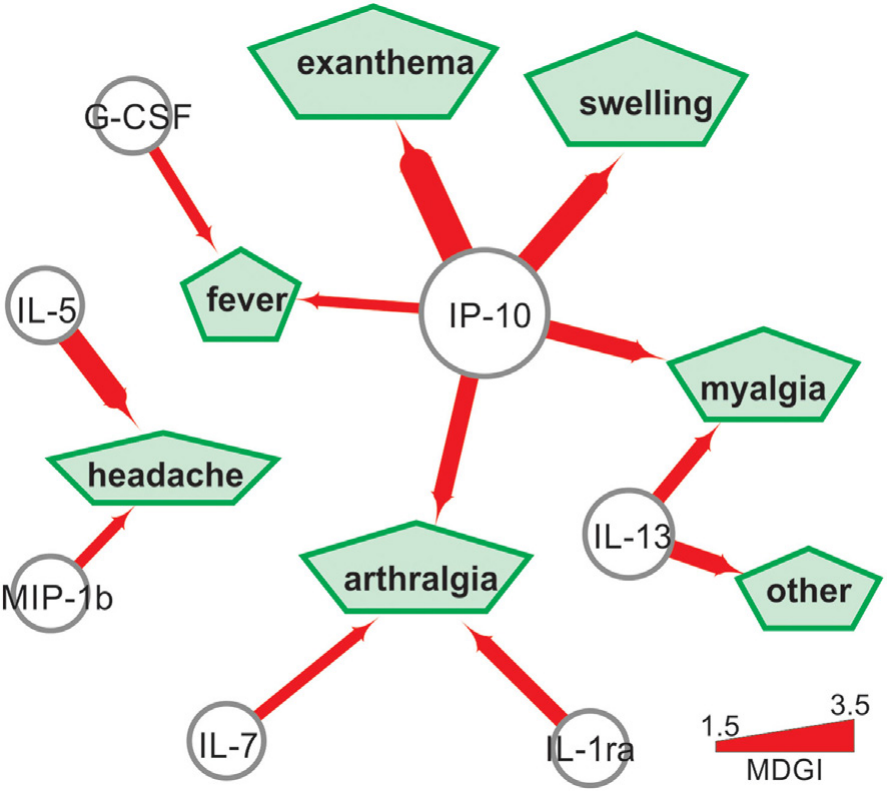
Cytokine levels can predict the clinical symptoms of Zika virus-infected subjects. The green pentagons represent clinical symptoms, the nodes represent cytokines and the edges represent the mean decrease of the Gini index (MDGI). Arrow thickness is proportional to the MDGI index.

We also used the Random Forest method (25) to assess the power of plasma cytokine levels to predict Zika symptoms. This method generates the predictive importance of each cytokine in classifying patients with or without a specific symptom. Using a mean decrease of Gini index > 1.5, we constructed a network showing the associations between cytokine levels and symptoms (Figure 5). IP-10 was strongly associated with exanthema, swelling, myalgia, arthralgia, and fever. Headache was predicted mainly by IL-5 and to a lesser extent by MIP-1β, while arthralgia was predicted by IL-1ra and IL-7. G-CSF predicted fever, and IL-13 and IP-10 and IL-13 and IP-10 also predicted myalgia (Figure 5).

### Cytokine Changes in the Acute and Recovery Phases

**Figure 6:**
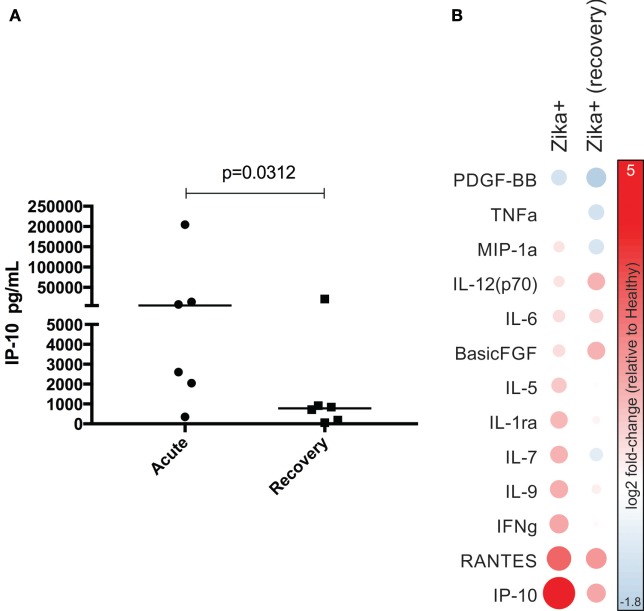
Comparison of plasma cytokine levels during the acute and recovery phases. **(A)** Cytokine measurements were performed in the plasma of six individuals in the recovery phase (2–3 weeks after the onset of symptoms) and compared to those performed during the acute phase. The dots on the graphs represent individuals. The Wilcoxon matched-pair test was used. *p*-Values are indicated at the top of the graph and were considered significant when *p* = 0.0312. **(B)** The levels of cytokines with fold-changes >2 over healthy control levels in the acute or recovery phases are represented. The node color represents the fold-change in cytokine levels between infected and healthy subjects.

To investigate whether cytokine, chemokine, and growth factor levels were altered during the recovery phase of ZIKV infection, we collected blood from six individuals 2–3 weeks after symptom onset. We then compared the cytokine levels in this group during acute and recovery phases, as well as with healthy controls. However, only IP-10 showed significantly lower levels in the recovery phase compared to the acute phase (*p* < 0.05, Figure 6A). Additionally, comparing the cytokine levels during acute or recovery phases of ZIKV-infected subjects with the levels in healthy controls, we observed decreases in IP-10, RANTES, IFN-γ, IL-9, IL-7, IL-1ra, and IL-5 in the recovery phase, and increases in IL-12-p70 and basic FGF (Figure 6B).

## Discussion

We aimed to determine the profile of systemic immune activation during acute ZIKV infection in residents of an endemic area and determine whether different symptoms were related to differential levels of specific cytokines/chemokines. We found that acute ZIKV infection induces a quite modest systemic immune activation, involving both proinflammatory and immunoregulatory cytokines and chemokines, compared to the healthy physiological state. It was surprising that out of 27 immune-active molecules studied, only 7 (26%) showed higher levels or detection frequencies in acute ZIKV infection, indicating that only a few of these molecules were dominantly mobilized. Cytokines and chemokines with higher levels during acute ZIKV infection were IP-10, RANTES, IFN-γ, IL-9, IL-7, IL-1ra, and IL-5, involving innate and adaptive immune responses. These cytokines have diverse functional patterns such as Th1 (IFN-γ), Th2 (IL-5), and Th9 (IL-9) cells, suggesting the activation of these T helper lymphocyte subsets in acute ZIKV infection. Other possible sources of chemokines and cytokines involved in early ZIKV infection are monocytes, dendritic cells, natural killer cells, and innate lymphocyte cells. Additionally, the higher levels of homeostatic/immunoregulatory cytokines, namely IL-7 and IL-1ra, indicate the simultaneous mobilization of immune molecules controlling inflammation.

Although the levels of only three dominantly proinflammatory molecules, IFN-γ, IP-10, and RANTES, were significantly higher in acute ZIKV infection in the endemic area, they appear to be sufficient for generating an effective antiviral response (26–28), once the subjects usually present viral clearance, at least in the peripheral blood and urine (29). These cytokines are closely related to each other, suggesting common pathways and synergic effects (30–32). Similar to our results, Kam et al. (33) found higher plasma levels of IFN-γ and IP-10 in acute ZIKV-infected subjects from another endemic area than in non-infected controls, reinforcing that ZIKV induces the production of these cytokines that can promote antiviral response and consequently a mild disease.

As monocytes are a significant source of IP-10 and RANTES in the peripheral blood, they are likely to be involved in early stage of infection. Recently, it has been shown *in vivo* and *in vitro* that blood monocytes, especially the CD14+CD16+ (intermediate) and CD14lowCD16+ (non-classical) subsets, are the primary ZIKV-infected cells in human blood (19, 20). It is believed that once activated, infected monocytes produce cytokines and migrate to different tissues promoting virus dissemination and most likely establishing virus reservoir.

Interestingly, in our study, the levels of IP-10 decreased in the recovery phase, time when usually the viremia is undetectable in plasma and urine (29). It is, therefore, possible that the higher levels of IP-10 reflect the presence of the virus in the peripheral blood or even in tissues or hidden reservoirs. However, whether IP-10 only controls the viral replication or also participates in immunopathogenesis of ZIKV infection is not yet known. Further studies are necessary to clarify the role of IP-10 in ZIKV infection. It is noteworthy that the excess of inflammatory responses can also play a deleterious role in clinical manifestations. Accordingly, our data show that IP-10 was strongly associated with exanthema, which occurs during the viremia. Consistently, high levels of IP-10 and IFN-γ have been shown to be involved in non-IgE-mediated hypersensitivity reactions, such as maculopapular exanthema (34, 35).

Headaches in ZIKV-infected individuals were associated with the presence of increased plasma TNF-α, IL-17A, IL-2, IL-9, IL-12p70, MIP-1α, G-CSF, GM-CSF, and VEGF, as well as significantly higher levels of IL-5, suggesting that these cytokines can play a role in the mechanisms underlying headache. Accordingly, high plasma levels of IL-5 (36), MIP-1α (37), and RANTES (38) have been reported in migraine sufferers, but their mechanistic contributions are yet to be investigated. Studies on inflammatory mediators in the pathophysiology of headache have shown the main action of IL-5 during migraine episodes (36, 39). Despite increased levels of IL-5 in ZIKV-infected individuals, we observed normal levels of eosinophils and IgE (Figure S2 in Supplementary Material), suggesting that exanthema, the most frequent symptom of our cohort (80.5%), may not involve eosinophils and IgE. This may be related to the day of symptom onset and the severity of the disease. In dengue infection, total IgE serum antibodies showed increased levels in severe cases starting 7 days after primary dengue infection (40–42). We cannot exclude the possibility that IgE levels increase later in the disease course.

Also interesting was the association between arthralgia and increased production of IL-1ra, a soluble inhibitor of the IL-1 receptor that inhibits the function of IL-1β, a dominantly proinflammatory cytokine in inflammatory diseases affecting the joints (43). Indeed, IL-1ra production has been observed in arthralgia, probably due to a negative feedback mechanism controlling inflammation (44, 45). Thus, it is likely that in acute-ZIKV infection, IL-1ra plays an immunoregulatory role, controlling inflammation. Fever, the third most reported symptom in acute ZIKV infection, was negatively associated with G-CSF. The mechanism by which G-CSF controls fever is unclear. However, this growth factor is commonly used in cancer patient treatment and consequently decreases fever, suggesting an antipyretic effect (46).

It was quite unexpected that many proinflammatory cytokines/chemokines, such as TNF-α and IL-6, were not significantly higher during acute ZIKV infection in an endemic area, in contrast with other viral infections, such as dengue (47). Our findings also contrast with a report on acute ZIKV infection in six individuals who were not from an endemic area, in which a more striking immunoactivation profile was observed, with higher levels of various inflammatory cytokines/chemokines, including IL-1β, IL-6, IL-17, MIP-1α, IP-10, RANTES, IFN-γ, and TNF-α, as well as some immunoregulatory cytokines such as IL-4, IL-10, and IL-13, and IL-2 and IL-9, which display both immunoregulatory and inflammatory functions (21). Although, a diverse profile of cytokines was observed in both studies, this marked difference in global systemic immune activation profile with acute ZIKV infection in residents and non-residents of endemic areas suggests that constant exposure to ZIKV and possibly other environmental factors may affect the immunological inflammatory impact of infection. In this context, it is important to point out that the endemic area of our study has a higher prevalence of arboviruses and relevant index of Yellow Fever vaccination (48, 49). Thus, we hypothesize that the mild ZIKV fever observed in the endemic population may be due to the immune crossreactivity among the arboviruses such as DENV, YFV, and ZIKV. In addition, we raise that this less inflammatory immune profile in our endemic area, though likely to be efficient in systemic virus clearance, could eventually have an unfavorable impact in contexts involving immunoregulatory physiological shift, such as in pregnancy.

In conclusion, our data indicate that acute ZIKV infection in a larger cohort resident to an endemic area displays a modest systemic immune activation profile, involving both proinflammatory and immunoregulatory cytokines and chemokines. Even though these cytokine and chemokines are modestly produced, they are associated with the mostly frequent symptoms such as exanthema, headache, arthralgia, and fever. The mild disease observed in ZIKV-infection can be a result of a balance between pro- and anti-inflammatory cytokines and chemokines that can contribute to virus control and clinical symptom association. Taken together, our results advance the understanding of essential molecules involved in the immunopathogenesis of acute ZIKV infection and highlight the occurrence of a differential immunologic profile in ZIKV infection in an endemic area.

## Ethics Statement

Ethical approval for this study was obtained from the Institutional Review Board from Pontifical Catholic University of Goias (CEP—Research Ethics Committee), under the protocol number 46073815.9.0000.00370. All study subjects signed a written informed consent form before the interview and blood collection in accordance with the Declaration of Helsinki.

## Author Contributions

SF and IP conceived and designed the study. JB, PS, and RK collected the samples, and JB and PS performed the experiments. PG-D performed the Random Forest analysis. HN analyzed the data using systems biology. SF, IP, VC, HN, JF, and PN wrote the manuscript. All authors reviewed and approved the manuscript.

## Conflict of Interest Statement

The authors declare that the research was conducted in the absence of any commercial or financial relationships that could be construed as a potential conflict of interest.

## Footnotes

^1^https://www.cdc.gov/zika/laboratories/lab-safety.html (Accessed: February 20, 2018).

^2^http://www.cytoscape.org/ (Accessed: November 10, 2017).

## Supplementary Material

Figure S1 Plasma cytokine concentrations in acute ZIKV-infected individuals compared to healthy controls. IFN-γ **(A)** and IL-10 **(B)** levels in the plasma of acute ZIKV-infected individuals and healthy donors were quantified using ELISA. The horizontal lines indicate the medians. The dots on the graphs represent individuals. P-values are indicated at the top of each graph. The non-parametric Mann-Whitney test was used, and differences were considered statistically significant when *p* < 0.05.

Figure S2 Quantification of total plasma IgE in ZIKV-infected individuals and healthy controls. Plasma IgE levels were measured using ELISA. **(A)** IgE levels in ZIKV-infected individuals and healthy controls. The Mann-Whitney test was used for statistical analysis. **(B)** Stratification according to the day of symptom onset. The Kruskal-Wallis test was performed, and p values were not significant (*p* < 0.05). OD, optical density.
