# Supplementary figures and images for: Acute Zika Virus Infection in an Endemic Area Shows Modest Proinflammatory Systemic Immunoactivation and Cytokine-Symptom Associations
Source: Front Immunol. 2018 May 3;9:821. doi: 10.3389/fimmu.2018.00821 (PMC5943559; doi:10.3389/fimmu.2018.00821)

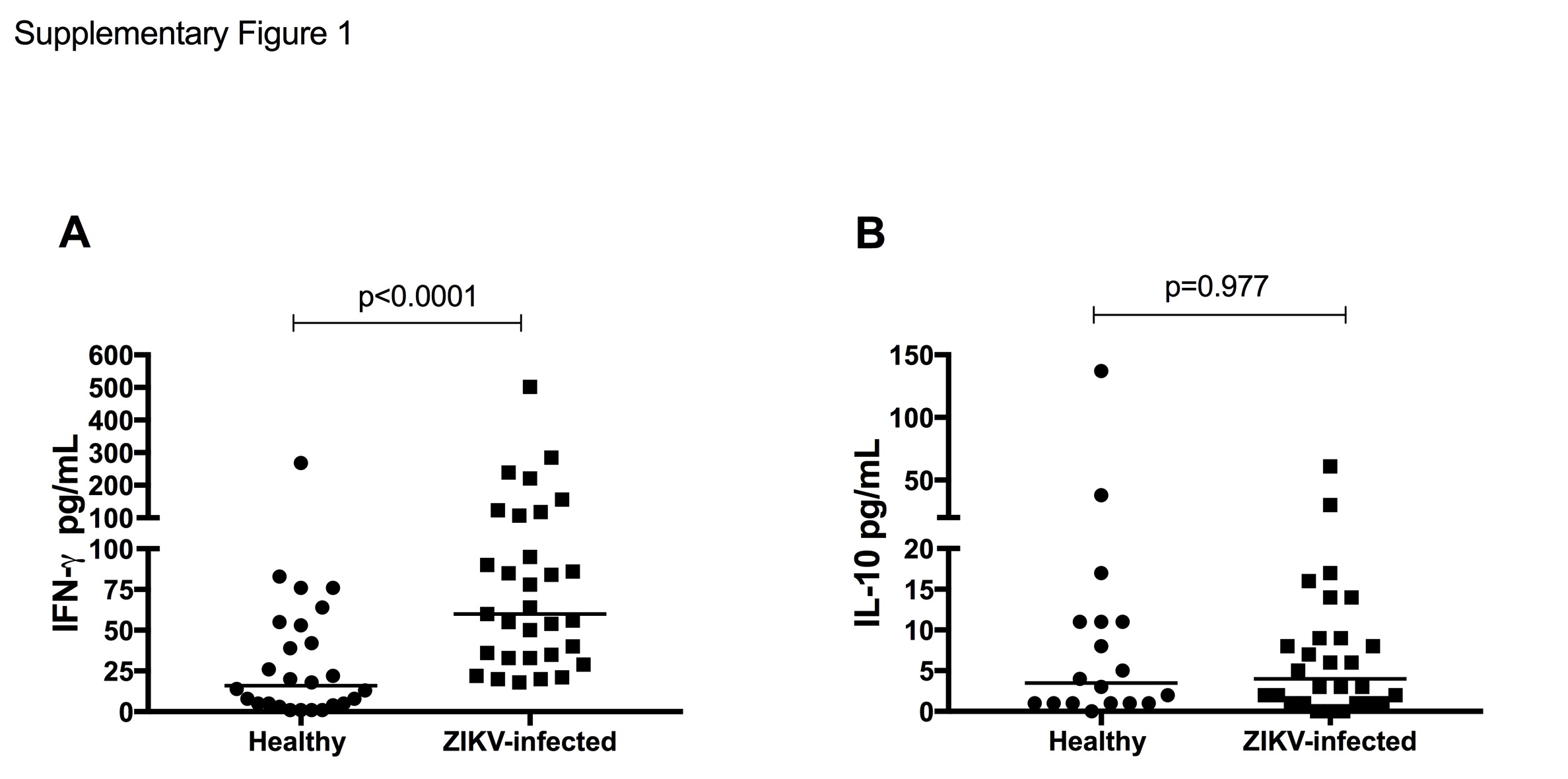

Supplement: Figure S1 — Plasma cytokine concentrations in acute ZIKV-infected individuals compared to healthy controls. IFN-γ (A) and IL-10 (B) levels in the plasma of acute ZIKV-infected individuals and healthy donors were quantified using ELISA. The horizontal lines indicate the medians. The dots on the graphs represent individuals. P-values are indicated at the top of each graph. The non-parametric Mann-Whitney test was used, and differences were considered statistically significant when p < 0.05. [file image_1.jpeg]

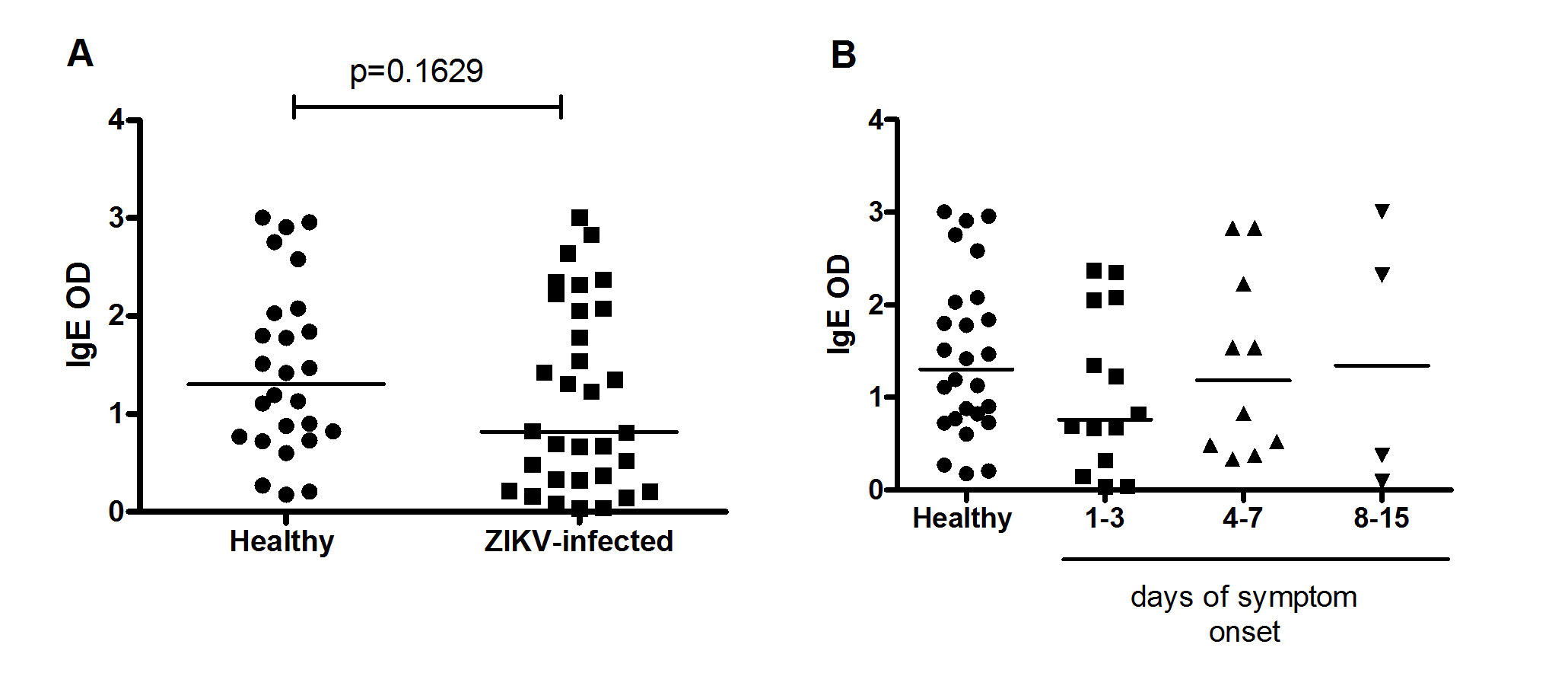

Supplement: Figure S2 — Quantification of total plasma IgE in ZIKV-infected individuals and healthy controls. Plasma IgE levels were measured using ELISA. (A) IgE levels in ZIKV-infected individuals and healthy controls. The Mann-Whitney test was used for statistical analysis. (B) Stratification according to the day of symptom onset. The Kruskal-Wallis test was performed, and p values were not significant (p < 0.05). OD, optical density. [file image_2.jpeg]
